# Construction of nested genetic core collections to optimize the exploitation of natural diversity in *Vitis vinifera *L. subsp. *sativa*

**DOI:** 10.1186/1471-2229-8-31

**Published:** 2008-04-02

**Authors:** Loïc Le Cunff, Alexandre Fournier-Level, Valérie Laucou, Silvia Vezzulli, Thierry Lacombe, Anne-Françoise Adam-Blondon, Jean-Michel Boursiquot, Patrice This

**Affiliations:** 1 UMR 1097 DIA-PC, Equipe « génétique Vigne », INRA-Supagro, 2 place Viala, F-34060 Montpellier, France; 2 IASMA Research Center, 38010 San Michele all'Adige (TN), Italy; 3 UMR 1165 INRA-CNRS-Université d'Evry Génomique Végétale, 2, rue Gaston Crémieux CP 5708, F-91057 EVRY cedex, France

## Abstract

**Background:**

The first high quality draft of the grape genome sequence has just been published. This is a critical step in accessing all the genes of this species and increases the chances of exploiting the natural genetic diversity through association genetics. However, our basic knowledge of the extent of allelic variation within the species is still not sufficient. Towards this goal, we constructed nested genetic core collections (G-cores) to capture the simple sequence repeat (SSR) diversity of the grape cultivated compartment (*Vitis vinifera *L. subsp. *sativa*) from the world's largest germplasm collection (Domaine de Vassal, INRA Hérault, France), containing 2262 unique genotypes.

**Results:**

Sub-samples of 12, 24, 48 and 92 varieties of *V. vinifera *L. were selected based on their genotypes for 20 SSR markers using the M-strategy. They represent respectively 58%, 73%, 83% and 100% of total SSR diversity. The capture of allelic diversity was analyzed by sequencing three genes scattered throughout the genome on 233 individuals: 41 single nucleotide polymorphisms (SNPs) were identified using the G-92 core (one SNP for every 49 nucleotides) while only 25 were observed using a larger sample of 141 individuals selected on the basis of 50 morphological traits, thus demonstrating the reliability of the approach.

**Conclusion:**

The G-12 and G-24 core-collections displayed respectively 78% and 88% of the SNPs respectively, and are therefore of great interest for SNP discovery studies. Furthermore, the nested genetic core collections satisfactorily reflected the geographic and the genetic diversity of grape, which are also of great interest for the study of gene evolution in this species.

## Background

The study of natural allelic diversity has proved fruitful in understanding the genetic basis of complex traits [1-6]. However, exploiting it successfully through association genetics requires basic knowledge of the extent of allelic variation within a species. One of the most interesting ways to achieve this goal consists of developing high-density diversity maps like the those developed in human and chicken, which allow the identification of causal polymorphisms for important traits [7-10]. The recent publication of the first high quality draft of the grapevine genome sequence opens the way to building such a diversity map [11]. Like in animals or in other perennial plant species where genetic approaches based on the study of segregating populations are hampered by a long biological cycle, association genetics is of particular interest in grapevine.

The development of diversity map relies on the discovery of sequence polymorphisms in the genome in a small set of genotypes that are as representative as possible of available genetic diversity. Such a concept was first proposed by Frankel and Brown under the name of core collection [12]. Core collections can be built using different types of markers. For example, molecular markers were used for rice, wheat and potato, while for yam a core collection was built using the origin of cultivars, eating quality, tuber shape, tuber flesh colour, and morphotype [13-16]. Different strategies have been proposed to assist the construction of core collections including the M-Method developed by Schoen and Brown and implemented in the software MSTRAT [17-20]. This strategy has been successfully used for the construction of core collections in *Arabidopsis thaliana *and *Medicago truncatula *and was also proposed as a preliminary step in association genetics [21-24].

Large collections of genetic resources are available for grapevine especially in Europe [25]. The largest one is held by INRA in France at the domain of Vassal: this collection contains 7000 accessions of *Vitis *genus of worldwide origin [26]. The genotyping of the whole collection using 20 well-scattered SSR markers is complete Laucou et al. (in prep). The cultivated compartment (*V. vinifera *L. subsp. *sativa*) is represented by 3900 accessions corresponding to 2262 unique genotypes (Laucou et al, in prep), from 38 different countries. It represents about a half of the known grapevine cultivars [27]. The Vassal collection was highly diverse for *V. vinifera *L. subsp. *sativa*, exhibiting a total of 326 alleles for the 20 SSRs markers with an average of 16.3 SSR alleles per locus (Laucou et al, in prep). Moreover a large proportion of these alleles (17%) were present at very low frequency (freq < 0.05%).

A first core collection (M-core) in grape was recently developed based on 50 morphological traits on 1759 accessions from the Vassal collection [28]. It was used for a preliminary study of the extent of linkage disequilibrium (LD) in *V. vinifera *L. as well as for association studies [28,29]. However, the size of the M-core (141 individuals) limits its use for the analysis of wide sequence diversity.

Here we present the use of the data set obtained by Laucou et al. (in prep) to develop four nested genetic core collections (G-cores) suitable for the search for allelic diversity. The ability of retaining the SSR genetic diversity using different sample sizes was studied and compared to the SSR diversity present in the M-core and in the Vassal collection. Finally, the allelic diversity captured at the sequence level in the different sub-cores was analysed by sequencing three gene fragments. This work provides the foundation required for the development of a detailed map of haplotypic diversity in grapevine.

## Results

### Construction of nested core collections representing the available germplasm diversity of cultivated V. vinifera L

**Figure 1:**
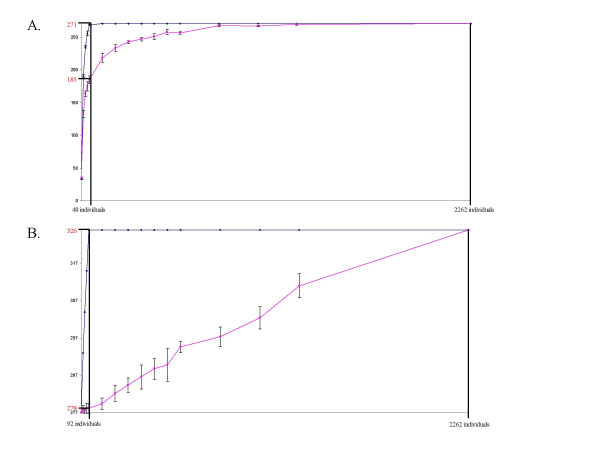
**Redundancy curves obtained using MSTRAT software**. Redundancy curves with standard deviation obtained using MSTRAT software (five independent samplings). Determination of the optimal size allowed the capture of all alleles of the original sample. A. For the 271 alleles of the restricted Vassal collection using the M-method (blue dot) and random sampling method (pink dot). B. For the 326 alleles of the Vassal collection using the G-48 core as core using the M-method (blue dot) and random sampling method (pink dot).

We first determined the optimal size of a core collection by retaining the 271 alleles showing a frequency above 0.05%. Forty-eight cultivars were necessary to capture 100% of the 271 alleles (Figure 1A). Within this core collection of 48 cultivars (G-48), we then determined the two most diverse samples of 12 (G-12) and 24 (G-24) cultivars (Table 1). In order to assess the robustness of these nested core collections, we calculated the percentage of identical varieties among the G-12, G-24 and G-48 core collections obtained in the second run using the same process, which corresponded to 83.3% (10) of the varieties selected in the two G-12 to 83.3% (20) of the varieties selected in the two G-24 and to 60.24% (29) of the varieties selected in the two G-48. Among these two sets of samples, the G-48 core collection presenting the highest Nei's index was chosen as the reference core collection (Table 2).

**Table 1:** SSR diversity within each sample of the G-core compared to the Vassal collection with and without the rare allele (Restricted Vassal collection).

Sample Name	Size	Number of alleles	Nei's indices	Observed heterozygosity	Percentage of total SSR diversity	Percentage of restricted SSR diversity	Correlation of SSR frequency with Vassal collection (R^2^)
Vassal collection	2262	326	0.76	0.75	100%	100%	
Restricted Vassal collection	2262	271	0.76	0.75	83%	100%	1
G-12 core	12	191	0.83	0.80	58%	70%	0.77
G-24 core	24	239	0.83	0.81	73%	88%	0.85
G-48 core	48	271	0.82	0.80	83%	100%	0.92
G-92 core	92	326	0.81	0.78	100%	100%	0.94
M-core	141	227	0.76	0.75	70%	81%	0.98

**Table 2:** Nested genetic core collection of 12 to 92 varieties.* Varieties bred from cultivars of different geographical origin: the countries listed are breeding locations.

Size	Variety name	Variety number	Country	Nbr of alleles
12	Tsolikouri	2668	Georgia	
12	Voskeat	2511	Armenia	
12	Kapistoni tétri hermaphrodite (Coll. Kichinev)	3242	Georgia	
12	Lameiro	3380	Portugal	
12	Médouar	3381	Israel	
12	Chirai obak	1186	Tajikistan	
12	Espadeiro tinto	1498	Portugal	
12	Araklinos	1805	Greece	
12	Plant du Maroc E (Coll. Meknès)	2158	Morocco	
12	César	225	France	
12	Orlovi nokti	2461	Russia	
12	Tsitsa Kaprei	2471	Moldavia	191

24	Variété d'oasis Bou Chemma 46	3281	Tunisia	
24	Uburebekur	3270	Romania	
24	Chouchillon	192	France	
24	Mehdik	2082	Iran	
24	Assyl kara	2505	Russia	
24	Pervenetz praskoveïsky	2651	Russia	
24	Pletchistik	2652	Russia	
24	Ak ouzioum tagapskii	2897	Kyrgyzstan	
24	Orbois	294	France	
24	Cabernet franc	324	France	
24	Katta-kourgan	556	Uzbekistan	
24	Kichmich tcherni	3264	Turkey	239

48	Tandanya faux	3279	Australia*	
48	Veltliner rot	284	Austria	
48	Yapincack faux	3292	Turkey	
48	Frühe Meraner	3183	Italy	
48	Kisilovy	3349	Russia	
48	Lumassina	3312	Italy	
48	Mourisco (Coll. EVV Amandio Galhano)	3379	Portugal	
48	Raisin banane noir	3384	Algeria	
48	Riesling bleu	3073	France	
48	Frappato di Vittoria	1318	Italy	
48	Tinto Cao	1488	Portugal	
48	Ag isioum	1563	Dagestan	
48	Orangetraube	1569	Germany	
48	Onusta	1980	Italy*	
48	Malvasia di Sardegna	2166	Italy	
48	Armenia	2267	Armenia*	
48	Jo Rizling	2563	Hungary*	
48	Krakhouna	2638	Georgia	
48	Portan	2796	France*	
48	Misguli kara	2917	Ukraine	
48	Bayadi du Liban	2998	Lebanon	
48	Bakarka	3008	Hungary	
48	Catanese nero	2398	Italy	
48	Retagliado bianco	67	Italy	271

92	Istchak rouge	3272	Uzbekistan	
92	Verdelho tinto	3205	Portugal	
92	Fantasy seedless	3051	USA*	
92	Kaisi baladi	3219	Syria	
92	Malahy	3238	Iran	
92	Koutlaksky belyi	3160	Ukraine	
92	Variété d'oasis Tozeur 17	3228	Tunisia	
92	Long Yan	3142	China	
92	Plant de Querol 98-N-2 (Coll. Torres SA)	3304	Spain	
92	Albarola rossa faux (Coll. Pisa)	3329	Italy	
92	Barbera selvatico del Grosseto	3320	Italy	
92	Doppel Augen	3151	Azerbaijan	
92	Duc de Magenta	819	France*	
92	Graeco	3224	Tunisia	
92	Lambrusco del Caset	3181	Italy	
92	Badagui	3156	Georgia	
92	Moscatel de Oeiras faux (Coll. Bordeaux)	3266	unknown	
92	Nero grosso	3176	Italy	
92	Agoumastos	3386	Greece	
92	Rich baba rose faux	3154	Russia	
92	Colorino	1353	Italy	
92	Uva de Rey	1395	Spain	
92	Tinta castellõa	1540	Portugal	
92	Alburla	1606	Ukraine	
92	Korithi aspro	1766	Greece	
92	Canner seedless	1833	USA*	
92	Agourane	1898	Algeria	
92	Morlin gris	2067	France	
92	Askari	2081	Iran	
92	Bogazkere	2104	Turkey	
92	Jeludovii	2253	Romania	
92	Tchilar	2274	Armenia	
92	Peygamber üzümü	2340	Turkey	
92	Lambrusco viadanese	2351	Italy	
92	Vernaccia di San Gimignano	2360	Italy	
92	Alexandroouli	2500	Georgia	
92	Malaga II (Dumas)	2570	France*	
92	Sapéré otskhanouri	2655	Georgia	
92	Khindogny	2664	Iran	
92	Yapincak	2768	Turkey	
92	Arna-guirna	2899	Azerbaijan	
92	Romorantin	304	France	
92	Mandilaria	341	Greece	
92	Mauzac faux de Cahuzac	357	France	326

The G-48 core was used as a core to build the final core collection retaining the 326 alleles found in the cultivated compartment of *Vitis vinifera *L. represented in the Vassal collection by Laucou et al. (in prep). The optimal size of this final core collection was 92 individuals (Fig. 1B). The cultivars added at this step contained only rare alleles (freq < 0.05%, present on less than 3 copies), which corresponded to less choice for the selection of varieties. Indeed, only two alternative samples were proposed by MSTRAT, with only one individual differing between the two samples: Rich baba rose faux *versus *Kizil. Again, we selected the G-92 presenting the highest value for the Nei's index as the reference core collection for the cultivated compartment of *V. vinifera *L; the resulting final core collection is listed in Table 2.

In order to estimate the gain of SSR allelic diversity, we compared the number of alleles captured in samples obtained by the M-method and by random sampling. In each case, when using the M-method, we observed a gain (Table 3), the greatest of which being obtained for the selection of the G-48.

**Table 3:** Gain obtained using the M-method at each step of the construction of the nested core collection *versus *random sampling.

Original collection	Sample size	M-method (mean number of alleles for 5 runs)	Random sampling (mean number of alleles for 5 runs)	Gain using M-method
Vassal with G-48 used as core	92 individuals	326	278.2 (+/- 1.3)	15%
Restricted Vassal collection (without rare alleles freq < 0.05%)	48 individuals	269.8 (+/- 1.6)	185.2 (+/- 5.7)	31%
G-48 (without rare alleles freq < 0.05%)	24 individuals	238.2 (+/- 0.4)	218.8 (+/- 6.5)	8%
G-24 (without rare alleles freq < 0.05%)	12 individuals	190.8 (+/- 0.4)	177.8 (+/- 1.6)	6%

### Analysis of the diversity retained in the nested core collections using different descriptors

The reference nested core collections for the cultivated compartment of *Vitis vinifera *were described for several characteristics and compared to the Vassal collection and to the M-core collection (141 individuals) defined by Barnaud et al. [28].

#### SSR diversity

The nested core collections represented 58% to 100% of the total SSR diversity of the Vassal collection and 70% to 100% of the restricted SSR diversity of the Vassal collection (only considering alleles with frequencies higher than 0.05%) (Table 1). All the SSR alleles with a frequency of more than 5% within the Vassal collection are present in the G-12 core and all those with a frequency of more than 3.5% within the Vassal collection are present in the G-24. The values of the unbiased Nei's diversity index and the level of unbiased observed heterozygosity for the G-12 core, G-24 core and G-48 core collection were quite similar and slightly higher than those calculated for the G-92 core collection. These values were slightly higher than those of the Vassal collection and of the M-core (Table 1). We also compared allele frequencies of the SSR markers in the three G-cores and in the M-core with the frequencies observed in the Vassal collection: the best correlation was obtained between the Vassal collection and the M-core (r2 = 0.98) and the G-92 (r2 = 0.92) core collections (Table 1).

#### Geographic origin and final uses

**Figure 2:**
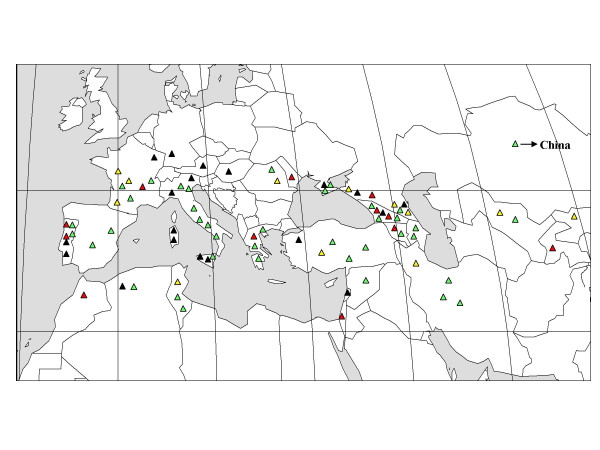
**Probable geographic origin of the varieties contained in the nested genetic core collections**. Each triangle corresponds to one variety, red triangles correspond to the first sub-sample of the nested genetic core collection (G-12), yellow triangles to the second sub-sample (G-24), black triangles to the third sub-sample (G-48) and green triangles to the fourth sub-sample (G-92). Ten varieties belonging to the Core G-92 did not have a precise geographical origin and are not shown on this map.

The definition of the true geographical origin of grapevine cultivars is sometimes difficult due to many migration events with humans [30]. Based on current knowledge, the cultivars held in the Vassal collection originated from 38 countries, with about half of them from Western Europe (France, Iberian Peninsula and Italy). The cultivars selected in the nested core collection originated from 27 different countries (Table 2). However 10 varieties of the G-92 sample could not be assigned to a precise geographical origin. Among them, 9 varieties were recent crosses between varieties from different countries (indicated by * in the Table 2) and one have an unknown origin (Moscatel de Oeiras faux). Moscatel de Oeiras faux microsatellite data seemed to indicate a Western Europe origin when compared to the whole collection. The origins of the 82 remaining varieties were well distributed (Figure 2): 21 (25%) came from the Caspian region (Dagestan, Georgia, Armenia and Azerbaijan) and the Middle East (Iran) which corresponds to the center of domestication, and 35 (42%) came from Western Europe and North Africa (Iberian Peninsula, Morocco, Algeria, Tunisia, Italy and France) (Table 4). Interestingly, five varieties (6% of the G-92) originated from Central Asia and Asia despite their very limited representation in the whole collection (less than 2%).

**Table 4:** Distribution of the geographical origin and the final use of the cultivars in the different samples

Region or Final uses	Western Europe and North Africa	Center of domestication	Asia and central Asia	Other area	Wine cultivars	Table cultivars	Wine and table cultivars
Vassal collection	56%	3%	1.6%	39.4%	55%	36%	9%
M-core	58%	7.2%	0.9%	33.9%	63%	30%	7%
G-12 core	33%	33%	8%	26%	67%	33%	0%
G-24 core	33%	33%	12.5%	21.5%	58.5%	37.5%	4%
G-48 core	37.5%	23%	6.25%	33.25%	56%	31%	12.5%
G-92 core	42%	25%	6%	27%	56%	32%	12%

No differences were observed between the M-core (22 countries) and the Vassal collection (Table 4) whereas all the G-cores differed from the Vassal collection. Indeed the number of cultivars from Western Europe and the center of domestication were more balanced in the G-92 core with a very good representation of the whole set of geographic origins. The same trend was observed in the different sub-cores (Table 4).

We also compared the G-92 core collection, the M-core and the Vassal collection with respect to the final use of the cultivars: wine making (wine cultivars), fruit consumption (table cultivars) or both (wine/table cultivars). The M-core and the different G-cores all resembled the Vassal collection (Table 4).

### Evaluation of the capture of unlinked diversity in the nested core collections

Next, we assessed the ability of the nested G-core samples to capture diversity unlinked to the SSR markers used to build the nested core collection. Barnaud et al. estimated using 38 SSR markers mapped on five different linkage groups (LG) with a maximum distance of 30 cM that LD in grape extends only within LG and is around 16.8 cM maximum [28]. We analysed the polymorphism of three gene fragments mapped further than 16.8 cM from the SSR markers in the same linkage group. *DFR *mapped in LG 18, 25.3 cM from the SSR marker VVIn16; *L-DOX *mapped in LG 8, 26 cM from the SSR marker VMC1b11 and *BURP *mapped in LG 3, 26 cM from the SSR marker VVMD28.

Forty-one nucleotide polymorphisms (40 substitutions and 1 in/del) were observed in the G-92, ranging from 12 to 15 depending on the gene fragment (Table 5). The total polymorphism is thus one SNP for 49 nucleotides. The number of SNPs per base also varied between the three gene fragments: one SNP for every 58 nucleotides for *DFR*, one SNP for every 42 nucleotides for *L-DOX *and one SNP for every 50 nucleotides for *BURP*. The difference of genetic diversity between coding and non coding region of the sequences was estimated only for the *DFR *sequence which has a quite similar length of the two types of regions. For this gene the polymorphism was different between coding and non-coding regions with a ratio of 3.2 (one SNP for every 127 nucleotides for coding region versus one SNP for every 39 nucleotides for non-coding region). Considering all genes together, the number of SNPs detected increased from 32 to 36 between the G-12 and the G-24 cores and from 36 to 40 between the G-24 and the G-48 cores. Only one more SNP was discovered in the G-92 core than in the G-48 core for the *L-DOX *gene fragment (this SNP is present in two varieties: Œil de Dragon and Badagui). The higher number of SNPs in the G-24 than in the G-48 cores was due to two genotypes: Yapincack with three additional SNPs in the *DFR *gene fragment and Kisilowy with one additional SNP in the *L-DOX *gene fragment.

**Table 5:** Number of polymorphic bases (SNP or insertion deletions found in the DNA fragments)

		Core collection studied							
					
Gene	Total size (exon size/intron size)	G-12	G-24	G-48	G-92	M core	Total number in exon	Total number in intron	Total number
*DFR *(gi 499017)	810 nt (380 nt/430 nt)	10	11	14	14	7	3	11	14
*L-DOX *(gi 22010674)	500 nt (459 nt/41 nt)	9	10	11	12	8	12	0	12
*BURP *(gi 22014825)	700 nt (700 nt/0 nt)	13	15	15	15	10	15	0	15
Total	2010 nt (1539 nt/471 nt)	32	36	40	41	25	30	11	41

Estimation of the ability to capture unlinked diversity of the G-24 core and G-12 core was performed by comparing their SNP diversity with SNP diversity in five random samples of 24 individuals in the G-48 core and 12 individuals in the G-24 core. The number of SNPs in the different random samples varied from 35 to 37 SNPs for the five random samples of 24 individuals and from 30 to 34 SNP for the five random samples of 12 individuals. In order to compare SNP distribution, we also calculated the unbiased Nei's index, which varied from 0.24 to 0.25 for the five random samples of 24 individuals and from 0.30 to 0.32 for the five random samples of 12 individuals. The unbiased Nei's index of the G-24 and G-12 cores was respectively 0.28 and 0.33.

Estimating the unlinked diversity within the whole Vassal collection (2262 cultivars) would have been very fastidious. Consequently we compared the capture of unlinked diversity in the nested core collections and in the M-core developed only on morphological traits. The total number of SNPs in the M-core (25 SNPs; Table 5) was smaller than in any of the nested G-core samples, even the G-12 core (32 SNPs; Table 5). Moreover, none of the SNPs observed in the M-core was new compared to those found in the nested core collections.

## Discussion

In the present work, we developed a set of nested core collections from the cultivated compartment of the Vassal collection, using the M-method and SSR diversity data obtained on 2262 unique genotypes. However, in this way we did not take into account the somatic variants present within *V. vinifera *L. cultivated germplasm. The usefulness of core collections is due to their ability to capture the diversity of the whole species. Even the smallest nested core collections were more efficient in capturing allelic diversity than the M-core with its 141 accessions. The Vassal collection, which formed the basis of this work, includes around 3900 cultivars which correspond to 2262 unique SSR genotypes from 38 countries, including from the main domestication area. This represents more than half the varieties found world wide [27]. A core collection developed from Vassal collection is thus of major interest for the scientific community, and thanks to the vegetative propagation ability of grape, could be easily multiplied and distributed.

### Construction of the core collections

The first result of our work is the fact that only a small number of cultivars (92 individuals, 4% of the Vassal collection) are needed to represent the whole diversity and an even smaller number of cultivars are needed to capture all the most frequent alleles (48 individuals, 2.1%). The comparison with other models is not easy, as they have different biological characteristics, the original collection did not reach the same global diversity of the species, and the analyses are seldom performed in the same way. Nevertheless, the core collections developed for *A. thaliana *(18%) or *M. truncatula *(31%) using the same method required higher percentages of individuals selected to represent all the genetic diversity [21,22]. In our study we only considered the cultivated compartment which tends to be less diverse than wild compartments [31]. But the high level of heterozygosity of the grapevine is probably also one of the factors that allow a lower number of individuals than homozygous species like the two plant species mentioned above. Finally, the small number of individuals needed to represent the genetic diversity of the cultivated grapevine also pinpointed the high redundancy of the Vassal collection where many kingroups were highlighted and the interest in using such core collections to optimize the study of the phenotypic and genetic diversity in grapevine [32,33].

### Nested core collections are of great interest for identifying the sequence diversity that exists in the cultivated compartment of the V. vinifera species

The total genetic diversity revealed in the sequences of three gene fragments (2010 bp) in the G-92 core was quite high with 41 SNPs, i.e. one SNP for every 49 nucleotides. This is substantially higher than the level of genetic diversity observed in the M-core on the same gene set. Moreover, it is higher that the level of genetic diversity observed on an other set of 25 gene fragments totalling 12 kilobases sequenced on seven cultivated individuals (one SNP for every 118 nucleotides) by Salmaso et al. and on a set of 230 gene fragments, what represents the analysis of over 1 Mb of grape DNA sequence 11 grape genotypes (one SNP for every 64 nucleotides) by Lijavetzky et al. [34,35]. This comparison thus emphasises the interest of such a core collection for the discovery of genetic diversity.

Among cultivated species, polymorphism in grape is relatively high compared to *Zea mays *(one SNP every 100 nucleotides), *Pinus pinaster *(one SNP for every 102 nucleotides) and *Hordeum vulgare *(one SNP for every 78 nucleotides), while it is relatively low compared to wild species such as *A. thaliana *(one SNP for every 32 nucleotides) [21,36-38].

### G-48 core is highly diverse and non-redundant

The G-92 core was built taking into account extremely rare alleles. Considering the rapid evolution of SSR markers, we assumed that the alleles present in two cultivars or less in the collection did not adequately represent gene diversity and they were thus removed when we built the G-48 core [39-41]. Indeed, only one additional SNP was revealed in the G-92 sample (present in two cultivars and not in the M-core) compared to the G-48, thus validating our assumption. On one hand, the gain in the unlinked diversity was high in the G-48, probably due to the decrease in redundancy compared to the Vassal collection (revealed by the number of kingroups). On the other hand, when compared to a random sampling, the gain was much higher using the M-method. The final G-48 core is highly non-redundant and highly diverse. Moreover the G-48 core optimized the unlinked diversity in the three different regions sequenced compared to the M-core, whose individuals coming from Vassal collection were not selected based on their genotypes, by consequent they could be consider as a less optimized sampling within the Vassal collection.

The G-12 and G-24 cores already include respectively 78% and 88% of the SNPs markers present in the G-92 core (80% and 90% of the G-48 core). They also include 58% and 73% of all the SSRs markers identified within the Vassal collection, representing a gain of 6% to 8% compared to random sampling from the G-48 or G-24 core. From a technical point of view, the size of the G-12 and G-24 cores is better suited for high throughput genomic studies and consequently highly suitable for ambitious projects of SNP discovery.

### Geographic origin and final uses of the varieties within the G-core

Interestingly the nested core collections constructed in the present work reflect the distribution of grapes in Europe and around the Mediterranean Sea but with over-representation of the cultivars originating from the Caspian region and Middle East, and under-representation of the cultivars from Western Europe (Iberian peninsula, France and Italy) compared to the Vassal collection. We compared the SSR allele frequencies of the nested core collections and of the Vassal collection and found low correlations. This result further emphasizes the decrease in redundancy in the core collections compared with the Vassal collection, but also reflected the relative high number of cultivars originating from Western Europe in the Vassal collection, whereas the main domestication center is the Middle East [30]. These two regions may thus represent important sources of genetic diversity for the *V. vinifera *L. species. They represent the cradle of viticulture and the first migration of cultivars by Greeks and Etruscans, and a second domestication center in Western Mediterranean region [42]. Finally, despite their low representation in the Vassal collection, the presence of cultivars from Asia and Central Asia in the nested core collections could also indicate an underexploited center of diversification worthy of prospection and analysis.

The proportion of table varieties, wine varieties and table/wine varieties was very well conserved in the nested core collections compared to the M-core and to the Vassal collection. The distinction between these three categories of cultivars is based on morphological traits such as berry size, bunch size and compacity but also on other traits such as the sugar/acid balance at maturity [43]. Previous studies have shown that there is strong genetic differentiation between these three groups of varieties that may be due either to divergent selection based on the same gene pool or to the use of specific gene pools for the development of the three types of varieties [44,27].

As a consequence, if the samples are well suited for analysis of allelic diversity, other uses can also be proposed for the cores, for example, the nested core collection could help understand the evolution of grape. Both G-12 and G-24 cores contained more frequent alleles representing ancient alleles while G-48 and G-92 may constitute subsequent diversification of cultivars in recent periods.

## Conclusion

In the present work, we developed a set of robust nested core collections of *V. vinifera *L. (cultivated compartment) that will facilitate the discovery of allelic diversity by the scientific community. Moreover, this is an important basic tool for the development of projects of association mapping in grapevine. In conclusion, even if these nested core collections are statistically too small to study correlations between phenotype and nucleotide diversity, their use for preliminary tests of hypothesis will speed up the selection of suitable candidates (for example by discarding unsuitable candidates) and for SNP discovery. Due to the perennial nature of grape and the ease of vegetative propagation, these nested core collections could easily be disseminated worldwide for analyses (by simple request at request-vassal@supagro.inra.fr).

## Methods

### Plant material and DNA extraction

For each genotype of the four nested core collections, an accession of the Vassal collection (Domain de Vassal, Herault, France) was selected (Table 1) and a batch of young leaves was collected and lyophilized for long-term conservation. Lyophilized leaves were ground twice for 1 min at 20 Hz using a Qiagen-Retsch MM300 crusher. DNA was extracted using the Qiagen DNeasy Plant mini kit (Qiagen) following the manufacturer's instructions with minor modifications: addition of 1% w/v of PVP-40 to the AP1 solution, addition of 180 μl AP2 instead of 130 μl and an additional step of 10 minutes centrifugation at 6000 rpm after incubation on ice, which enabled the majority of the cellular remains and aggregates formed after the addition of AP2 to be pelleted.

### Methods for the construction of the core collection

The dataset obtained by Laucou et al. (in prep) on the 2262 unique genotypes from the Vassal collection was used. The M-method proposed by Schoen and Brown and implemented in the MSTRAT software by Gouesnard at al. was used to generate the nested genetic core collections that maximize the number of observed alleles in the SSR data set [19,20]. The efficiency of the sampling strategy was assessed by comparing the total number of alleles captured using MSTRAT in samples of increasing size with the number of alleles captured in randomly chosen collections of the same size (five independent samplings). After having determined the optimal size of the nested core collections, 200 core collections were generated independently for each sample size. Putative core collections exhibiting the same allelic richness (determined by the total number of alleles represented) were ranked using Nei's index as the second criterion [45].

### PCR primer design

The gene sequences that were analysed were derived from three genes located on three separate chromosomes (Table 6). Two were involved in the anthocyanin metabolic pathway: the dihydroflavonol 4-reductase (*DFR, *gi 499017) present in one copy in the genome of *V. vinifera *L. and the leucoanthocyanidin dioxygenase (*L-DOX *gi 22010674) present at least in three copies in the genome of *V. vinifera *L. based on the NCBI database. The third gene codes for a BURP domain protein presenting a differential expression in a natural mutant of berry development compared to the wild type (*VvBURP1*; gi 22014825) [46-48]. Specific PCR primers (Table 2) were designed for the amplification of fragments of these three genes using Primer3 software and tested for amplification on the genomic DNA of the 12 individuals of the core G-12 [49].

**Table 6:** Localisation of the genes chosen for partial re-sequencing, specific PCR primers used and size of the gene fragment re-sequenced

DNA fragment (GenbanK)	LG located	Size	Primer forward (5'→3' sequence)	Primer reverse (5'→3' sequence)
*DFR *(X75964)	18	810 nt	CAAGCTGCATGGAAGTATGC	TTGGGCCATTCCGTTTTATTA
*L-DOX *(BQ795708)	8	500 nt	TTGAGCCCAATCATATTAGTTCC	GTGGCATGACCATTCTCCTC
*BURP *(BQ799859)	3	700 nt	CGAAAAGGGACACACAGAG	GTTCAGAGTAGGCCTCGGAA

Total		2010 nt		

### PCR amplification, sequencing, sequence analysis and SNP detection

The 25 μl PCR reaction mixtures contained 20 ng of genomic DNA, 50 mM KCl, 10 mM TRIS-HCl (pH 8.3), 0.4 mM of each primer, 125 μM of each dNTP, 1.5 mM MgCl2 and 2.5 U of Taq polymerase (Qiagen). PCR amplifications were performed in a MJ Research PTC 100 Thermal Cycler programmed as follows: 5 min denaturation at 94°C, 35 cycles of 94°C for 30 s, 52°C for 45 s, and 72°C for 1 min, followed by an extension step at 72°C for 8 min. The PCR products were purified using the Agencourt AMPure method (Beckman Coulter) and directly sequenced in the two ways using the Big Dye Sequencing kit according to the manufacturer's specifications (Applied Biosystems Inc.). The sequence products were purified using the Agencourt CleanSEQ method (Beckman Coulter) and loaded onto an ABI PRISM^® ^3130 XL (Applera) capillary sequencer. The DNA sequences were analysed using the Staden Package [50]. Heterozygous SNPs were identified as double pics on the chromatograms and coded according to international codes (nucleotide codes of the International Union of Biochemistry). Insertion/Deletions were easily identified by overlapping sequences. Sequencing both strands enable to deal with such events. Only SNPs present on both forward and reverse sequences were validated.

### Statistical analysis

Different indices were used in this study. The selection of reference core collections among those constructed using MSTRAT and exhibiting the same allelic richness (determined by the total number of alleles represented) was performed using Nei's index (Nei, 1987) as the second criterion. Nei's index is given for one locus by: ***I_Neij _***= ***1-*∑*p_ij_^2 ^***where ***pij ***represents the ***i ***allele frequency of the ***j ***locus. The Nei diversity index for all the loci is the sum of indices for each locus given by ***I_Nei _***= ∑***_j _I_Neij_^2^***. The more the allelic frequencies are equilibrated within a sample, the higher the value of Nei's index

As the samples compared were of different size (M-core, nested core collections and the whole collection) the comparison was performed using the unbiased observed heterozygosity and the unbiased Nei's index [45]. The unbiased Nei's index for the locus ***j ***is given by: ***H_Neij _***= ***(2n/2n-1) (1-*∑*p_ij_^2^) ***where ***n ***represents the number of individuals and where ***pij ***represents the ***i ***allele frequency of the ***j ***locus, the unbiased Nei diversity index for all the loci studied is given by ***H_Nei _***= ***(1/C) *∑*_j_H_Neij_^2 ^***where ***C ***is the number of loci studied. The more the allelic frequencies are equilibrated within a sample, the higher the value of the unbiased Nei's index. The observed heterozygosity for the ***j ***locus is given by ***H_obsj _***= ***1-*∑*x_ij_^2 ^***where ***x_ij _***represents the homozygote frequency for ***i ***allele of the ***j ***locus. The unbiased observed heterozygosity for the ***j ***locus is: ***H_unobsj _***= ***(2n/2n-1) (1-*∑*x_ij_^2^) ***>where ***n ***represents the number of individuals and the unbiased observed heterozygosity for all loci studied is ***H_unobs _***= ***(1/C) *∑*_j_H_unobsj_^2 ^***where ***C ***is the number of loci studied.

We compared the SSR frequencies found in the M-core and the nested G core-collections with those of the Vassal collection using the R^2 ^correlation coefficient. R^2 ^is given by ***R^2 ^***= ***(Cov_ij_/σiσj)^2 ^***where ***Covij ***is the covariance between the two samples compared and ***σi ***and ***σj ***are the variance of samples ***i ***and ***j ***respectively.

## Authors' contributions

LLC carried out the sequence, participated in the sequence alignment, performed the statistical analysis and drafted the manuscript. AF-L carried out the sequence and participated in the sequence alignment. VL carried out the SSR analysis. SV carried out the sequence. TL participated in the design of the study and performed the ampelographic analysis. A-FA participated in the design and coordination of the study and helped to draft the manuscript. J-MB participated in the design of the study and performed the ampelographic analysis. PT conceived of the study, participated in the design and coordination of the study and helped to draft the manuscript. All authors read and approved the final manuscript
